# Endoscopic Ultrasound

**DOI:** 10.1155/2013/508082

**Published:** 2013-01-10

**Authors:** Salem Omar, Manoop S. Bhutani, Kenjiro Yasuda, Tiing Leong Ang

**Affiliations:** ^1^Division of Gastroenterology, Department of Medicine, Faculty of Medicine, University of Malaya, 50603 Kuala Lumpur, Malaysia; ^2^Division of Internal Medicine, The University of Texas MD Anderson Cancer Center, Houston, TX 77030-1409, USA; ^3^Department of Gastroenterology, Kyoto Second Red Cross Hospital, 355-5 Haruobi-cho, Kamigyo-ku, Kyoto 602-8026, Japan; ^4^Department of Gastroenterology, Changi General Hospital, 2 Simei Street 3, Singapore 529889

There is now considerable interest in the clinical practice of EUS. Over the decades, EUS has evolved from diagnostic imaging and tissue acquisition to EUS-guided interventions. Technological advances have improved the EUS image quality and in recent years, image enhancement using contrast and elastography have also become more widely available. In this special issue that focuses on EUS, five papers explored various diagnostic and therapeutic aspects of EUS. Three papers examined the impact of EUS in cancer T-staging. Two other papers dealt with the issue of EUS-guided therapeutics. One explored the role of EUS-guided radiofrequency ablation (RFA) in an animal model while the other are concerned with EUS-guided biliary access and drainage. 

 In the first paper entitled “*Accuracy of endoscopic ultrasonography for determining the treatment method for early gastric cancer,*” K. Mandai and K. Yasuda examined the value of EUS in determining the therapeutic strategy for early gastric cancer. They found excellent correlation (92.8%) between EUS assessment of mucosa/sm1 involvement and histology. However the accuracy was reduced in the context of ulcerated lesions and lesions larger than 2 cm. 

In the second paper entitled “*Comparison of diagnostic accuracies of various endoscopic examination techniques for evaluating the invasion depth of colorectal tumours,*” S. Haruki et al. assessed the clinical value of magnifying endoscopy combined with EUS for estimating the invasion depth of colorectal tumours. They concluded that when it was difficult to evaluate the invasion depth of colorectal tumours on conventional endoscopy alone, the additional use of EUS may enhance diagnostic accuracy. 

In the third paper entitled “*Endoscopic ultrasound-guided radiofrequency ablation (eus-rfa) of the pancreas in a porcine model,*” S. Gaidhane et al. evaluated the use of a prototype monopolar probe inserted through the EUS-needle to perform EUS-RFA in the porcine pancreas. They found that EUS-guided RFA of the pancreatic head was well tolerated with minimal amount of pancreatitis. Recent studies have shown the feasibility of RFA in patients with stage III pancreatic cancer in open, percutaneous, or laparoscopic setting. This study offers the promise of a less invasive means of performing RFA of unresectable pancreatic cancer. 

In the fourth paper entitled “*Evaluation of endoscopic ultrasound image quality is necessary in endosonographic assessment of early gastric cancer invasion depth,*” S. Yamamoto et al. evaluated whether EUS image quality affected the accuracy of diagnosing the vertical invasion depth of early gastric cancer. They reported that low-quality EUS images led to an incorrect diagnosis of invasion depth of early gastric cancer, independent of tumour location and size. 

In the fifth paper entitled “*The spectrum of endoscopic ultrasound intervention in biliary diseases: a single centre's experience in 31 cases,*” S. Attasaranya et al. described the spectrum and experience of EUS-guided interventions in biliary diseases in a single-tertiary center. The overall technical success for EUS-guided biliary drainage was 77%; this was considerably higher in the last 2 years compared to the first 3 years (89% versus 61.5%), reflecting the effect of a learning curve in this challenging procedure, which may be associated with significant morbidity. 

